# Supplementary material for: Parietal alpha frequency shapes own-body perception by modulating the temporal integration of bodily signals
Source: Nat Commun. 2026 Jan 12;17:53. doi: 10.1038/s41467-025-67657-w (PMC12796323; doi:10.1038/s41467-025-67657-w)
Supplement: Supplementary file 2 — Reporting Summary [file 41467_2025_67657_MOESM2_ESM.pdf]

nature portfolio

Corresponding author(s): Mariano D'Angelo

Last updated by author(s): Nov 14, 2025

Reporting Summary

Nature Portfolio wishes to improve the reproducibility of the work that we publish. This form provides structure for consistency and transparency in reporting. For further information on Nature Portfolio policies, see our Editorial Policies and the Editorial Policy Checklist.

Statistics

For all statistical analyses, confirm that the following items are present in the figure legend, table legend, main text, or Methods section.

n/a	Confirmed	
☐	☒	The exact sample size (n) for each experimental group/condition, given as a discrete number and unit of measurement
☐	☒	A statement on whether measurements were taken from distinct samples or whether the same sample was measured repeatedly
☐	☒	The statistical test(s) used AND whether they are one- or two-sided<br>*Only common tests should be described solely by name; describe more complex techniques in the Methods section.*
☐	☒	A description of all covariates tested
☐	☒	A description of any assumptions or corrections, such as tests of normality and adjustment for multiple comparisons
☐	☒	A full description of the statistical parameters including central tendency (e.g. means) or other basic estimates (e.g. regression coefficient) AND variation (e.g. standard deviation) or associated estimates of uncertainty (e.g. confidence intervals)
☐	☒	For null hypothesis testing, the test statistic (e.g. F, t, r) with confidence intervals, effect sizes, degrees of freedom and P value noted<br>*Give P values as exact values whenever suitable.*
☒	☐	For Bayesian analysis, information on the choice of priors and Markov chain Monte Carlo settings
☒	☐	For hierarchical and complex designs, identification of the appropriate level for tests and full reporting of outcomes
☐	☒	Estimates of effect sizes (e.g. Cohen's d, Pearson's r), indicating how they were calculated

Our web collection on statistics for biologists contains articles on many of the points above.

Software and code

Policy information about availability of computer code

Data collection	Visuotactile stimuli were delivered using two robotic arms controlled with a custom made Python script. We recorded participants' verbal responses on Excel sheets. EEG signal was recorded on a dedicated laptop through BioSemi ActiveTwo software
Data analysis	EEG Data were analyzed using Matlab (R2023b, MathWorks, Inc), including EEGLAB, Brainstorm, and custom code. Behavioural data were analyzed using JASP (Version 0.13.03) and OriginLab (2019b). The code for the EEG analysis has been deposited in the Open Science Framework (OSF: https://osf.io/ytga5/overview), as well as the code for the Bayesian computational modeling (OSF: https://osf.io/s5p4v/overview)

For manuscripts utilizing custom algorithms or software that are central to the research but not yet described in published literature, software must be made available to editors and reviewers. We strongly encourage code deposition in a community repository (e.g. GitHub). See the Nature Portfolio guidelines for submitting code & software for further information.

Data

Policy information about availability of data

All manuscripts must include a data availability statement. This statement should provide the following information, where applicable:

- Accession codes, unique identifiers, or web links for publicly available datasets
- A description of any restrictions on data availability
- For clinical datasets or third party data, please ensure that the statement adheres to our policy

The aggregated psychophysical and preprocessed EEG data generated in this study have been deposited in the Open Science Framework (OSF: https://osf.io/ytga5).

Source data are provided in this paper. The code for the EEG analysis has been deposited in the Open Science Framework (OSF: https://osf.io/ytga5/overview), as well as the code for the Bayesian computational modeling (OSF: https://osf.io/s5p4v/overview)

Research involving human participants, their data, or biological material

Policy information about studies with human participants or human data. See also policy information about sex, gender (identity/presentation), and sexual orientation and race, ethnicity and racism.

Reporting on sex and gender	We collected gender information (self-reported) with the aim of ensuring gender-balanced samples for each experiment. None of our hypotheses were focused on gendere-related differences, therfore we did not include this information in any of our analyses.
Reporting on race, ethnicity, or other socially relevant groupings	None of the variables defined in our study hold social relevance in this context.
Population characteristics	In Experiment 1, we recruited 34 participants (19 females; ages 21–37 years; mean age 28.27 years). In Experiment 2, we recruited 57 participants (31 women; ages 18–43 years; mean age 27.70 years). In Experiment 3, we recruited 37 participants (20 women; ages 18–40 years; mean age 26.43 years).
Recruitment	Participants were recruited through a Swedish online platform for cognitive studies (Accindi.se), online student groups on Facebook, and flyers posted at the Biomedicum building in Solna. The advertisement included information about the stimuli used in the study, the equipment involved (robotic arms, EEG, or brain stimulation), compensation, and requirements to sign up (age range, no history of neurological or psychiatric disorders). No information about the hypothesis or analyses was included. Before confirming their participation, they were contacted via email to confirm that they met the criteria.
Ethics oversight	Swedish Ethical Review Authority

Note that full information on the approval of the study protocol must also be provided in the manuscript.

Field-specific reporting

Please select the one below that is the best fit for your research. If you are not sure, read the appropriate sections before making your selection.

☐ Life sciences ☒ Behavioural & social sciences ☐ Ecological, evolutionary & environmental sciences

For a reference copy of the document with all sections, see nature.com/documents/nr-reporting-summary-flat.pdf

Behavioural & social sciences study design

All studies must disclose on these points even when the disclosure is negative.

Study description	The three experiments included in this study were quantitative. The experiments featured a psychophysical component with a within-subjects design. In Experiment 1, two robotic arms applied touches to participants' hands and a rubber hand, either synchronously or at different delays. In detection-like tasks, participants were asked to indicate whether they felt that the fake hand seemed like their own hand or not (body ownership judgments). In another task, participants judged whether two briefly presented paired stimuli—a light and a small vibration—were synchronized or not (simultaneity judgments). Experiment 2 comprised the EEG component and also included similar judgment tasks, with the difference that, in simultaneity judgments, participants had to judge whether the touches on their own hand and on the fake hand were synchronous or not. Experiment 3 comprised the brain stimulation component. In a within-subjects design, participants underwent three experimental sessions, completing the same tasks under three different brain stimulation conditions.
Research sample	We recruited a total of 128 participants across three experiments. Participants were recruited through a Swedish online platform for cognitive studies (Accindi.se), online student groups on Facebook, and flyers posted at the Biomedicum building in Solna. To be eligible to participate, volunteers needed to be between 18 and 45 years old and have no history of neurological or psychiatric disorders. We collected only self-reported data on age and gender. In Experiment 1, we recruited 34 participants (19 females; ages 21–37 years; mean age 28.27 years). In Experiment 2, we recruited 57 participants (31 women; ages 18–43 years; mean age 27.70 years). In Experiment 3, we recruited 37 participants (20 women; ages 18–40 years; mean age 26.43 years).
Sampling strategy	Random sampling was conducted from volunteers who registered for study participation. Participants with a particular interest in EEG or bodily self-awareness may have been more likely to take part (self-selection bias). However, we do not believe this would affect our results.<br><br>The sample size was determined based on previous rubber hand illusion and EEG studies and power analyses (see Methods section). We aimed for 30 participants in Experiments 1 and 3 and 46 participants in Experiment 2. Since the rubber hand illusion is a prerequisite for performing the body ownership judgment task, participants were first tested on a classical rubber hand illusion paradigm. Those who did not experience a clear and reliable illusion were excluded in line with previous psychophysics studies. Recruitment continued until the target sample size was reached
Data collection	Data collection was conducted in the dedicated psychophysics and EEG testing room of the Brain, Body, and Self Laboratory at Biomedicum, Karolinska Institutet., employing two custom-made robotic arms to apply touches with millisecond precision, a computer to control the robot, a laptop to record participants' responses, an EEG system with a laptop to receive and record the EEG signal (Experiment 2), and a transcranial alternating current stimulation system. Prior to the experiment, all participants were

	required to read and sign a consent form. Participants were briefed on the task and underwent a screening session to evaluate whether they perceived the rubber hand illusion (see Sampling Strategies). In the EEG experiment, electrode placement occurred after participants completed the screening and signed the consent form. The room was lit, and participants were instructed to gaze at the rubber hand, while receiving touches on their own hidden hand
Timing	Data collection for Experiment 1 took place in July 2021. Data collection for Experiment 3 took palce between February and April 2022. Data collection for Experiment 2 took place between March and December 2023
Data exclusions	Participants who did not meet the inclusion criterion were excluded from the main experiment (see Sampling Strategy). In Experiment 1, four participants did not meet the inclusion criterion of being able to experience the rubber hand illusion. In Experiment 2, eleven participants did not meet the inclusion criterion. Finally, in Experiment 3, seven participants did not meet the inclusion criterion.
Non-participation	No participants withdrew or declined partecipation.
Randomization	Each participant completed all conditions as the experiments in our study employed a within-subject design. However, the order of tasks and conditions was pseudorandomized across participants.

Reporting for specific materials, systems and methods

We require information from authors about some types of materials, experimental systems and methods used in many studies. Here, indicate whether each material, system or method listed is relevant to your study. If you are not sure if a list item applies to your research, read the appropriate section before selecting a response.

Materials & experimental systems

n/a	Involved in the study
☒	☐ Antibodies
☒	☐ Eukaryotic cell lines
☒	☐ Palaeontology and archaeology
☒	☐ Animals and other organisms
☒	☐ Clinical data
☒	☐ Dual use research of concern
☒	☐ Plants

Methods

n/a	Involved in the study
☒	☐ ChIP-seq
☒	☐ Flow cytometry
☒	☐ MRI-based neuroimaging

Plants

Seed stocks	NA
Novel plant genotypes	NA
Authentication	NA
